# The BH3 mimetic drug ABT-737 induces apoptosis and acts synergistically with chemotherapeutic drugs in thyroid carcinoma cells

**DOI:** 10.1186/s12935-016-0303-8

**Published:** 2016-04-02

**Authors:** Martina Broecker-Preuss, Nina Becher-Boveleth, Stefan Müller, Klaus Mann

**Affiliations:** Department of Endocrinology and Metabolism, Division of Laboratory Research, University Hospital Essen, Hufelandstr. 55, 45122 Essen, Germany; Department of Nuclear Medicine, University Hospital Essen, Hufelandstr. 55, 45122 Essen, Germany; Department of Clinical Chemistry, University Hospital Essen, Hufelandstr. 55, Essen, Germany; Clinic of Nuclear Medicine, University Hospital Essen, Hufelandstr. 55, Essen, Germany; Center of Endocrinology Alter Hof München, Dienerstr. 12, Munich, Germany

**Keywords:** Apoptotic cell death, Dedifferentiated thyroid carcinoma, BCL-2 inhibitor, Thyroid cancer

## Abstract

**Background:**

Patients with dedifferentiated and anaplastic thyroid carcinomas that do not take up radioiodine are resistant to chemotherapeutic treatment and external irradiation and thus are difficult to treat. Direct induction of apoptosis is a promising approach in these apoptosis-resistant tumor cells. The BH3 mimetic ABT-737 belongs to a new class of drugs that target anti-apoptotic proteins of the BCL-2 family and facilitate cell death. The purpose of this study was to investigate the effect of ABT-737 alone or in combination with chemotherapeutic drugs on thyroid carcinoma cell lines.

**Methods:**

A total of 16 cell lines derived from follicular, papillary, and anaplastic thyroid carcinomas were treated with ABT-737. Cell viability was measured with MTT assay. Cell death was determined by cell cycle phase distribution and subG1 peak analyses, determination of caspase 3/7 activity and caspase cleavage products, lactate dehydrogenase (LDH) liberation assays and LC3 analysis by western blot.

**Results:**

The number of viable cells was decreased in all cell lines examined after ABT-737 treatment, with IC50 values ranging from 0.73 to 15.6 μM. Biochemical markers of apoptosis like caspase activities, caspase cleavage products and DNA fragmentation determined as SubG1 peak were elevated after ABT-737 treatment, but no LC3 cleavage was induced by ABT-737 indicating no autophagic processes. In combination with doxorubicin and gemcitabine, ABT-737 showed synergistic effects on cell viability.

**Conclusions:**

With these experiments we demonstrated the efficacy of the BH3 mimetic drug ABT-737 against dedifferentiated thyroid carcinoma cells of various histological origins and showed synergistic effects with chemotherapeutic drugs. ABT-737-treated cells underwent an apoptotic cell death. ABT-737 and related BH3 mimetic drugs, alone or in combination, may thus be of value as a new therapeutic option for dedifferentiated thyroid carcinomas.

## Background

Follicular cell derived thyroid cancer is the most common malignant endocrine neoplasm and can be classified into well differentiated thyroid cancer subtypes (about 90 %) and poorly differentiated or anaplastic subtypes (about 10 %) [1–3]. Of the differentiated carcinomas, 85–90 % are papillary thyroid carcinoma (PTC) and 10–15 % are follicular thyroid carcinoma (FTC). Most differentiated carcinomas are indolent tumors that progress slowly, and can be treated with thyroidectomy and radioiodine ablation [4]. However, in 10–15 % of patients initially diagnosed with differentiated carcinomas the tumor behaves aggressively and there is currently no effective treatment strategy since tumor dedifferentiation is accompanied by a reduction in radioiodine uptake and storage [4–6].

Anaplastic (undifferentiated) thyroid carcinomas (ATC) are highly aggressive and lethal tumors that depict highly infiltrative growth and have completely lost the ability to take up iodine [7]. Poorly differentiated thyroid carcinomas (PDTC) represent an intermediate between ATC and well-differentiated thyroid carcinomas with reduced ability of radioiodine uptake [8]. Besides their aggressive growth, the loss of the ability to take up radioiodine makes both PDTC and ATC difficult to treat, and contributes to the poor patient’s prognosis. Conventional chemotherapeutic treatment is ineffective against aggressive thyroid carcinomas [9, 10] and points to the inability of dedifferentiated thyroid cancer cells to undergo chemotherapy-induced cell death [11]. Facilitating cell death induction in these carcinoma cells thus is one new therapeutic option.

Resistance to cell death and the imbalance between cell division and cell death pathways contributes to uncontrolled tumor proliferation and is one characteristic feature of cancer cells and contributes to the “hallmark of cancer cells” [12]. Therapies that restore the ability of tumor cells to undergo apoptosis therefore are a promising new treatment opportunity.

In general, cell death can be induced by different signalling pathways and it morphologically and biochemically mainly appears as apoptosis, necrosis, necroptosis, and autophagy (review: [13]). According to the Nomenclature Committee on Cell Death [14], apoptosis is a form of regulated cell death characterized by the activation of caspases, a family of cysteine proteases [15]. Caspase activation results in degradation of various intracellular substrates, the fragmentation of nuclear DNA, and, in turn, to characteristic morphological changes of the affected cells [14]. Cells undergoing necrosis show cell swelling, early plasma membrane permeabilisation followed by cell rupture and the release of cellular material (review: [16]). The regulated form of necrosis is called necroptosis [17]. Autophagy at the other hand is characterized by self-digestion and thus recycling of cellular components. Thus, autophagy is also a survival mechanism for the whole cell population in situations like starvation or cellular damage (review: [18]).

Apoptosis is initiated by two signalling pathways, the extrinsic or death receptor pathway which is activated by binding of extracellular ligands of the tumor necrosis factor family to death receptors, and the intrinsic or mitochondrial pathway, which is regulated by proteins of the B-cell lymphoma (BCL-2) family (review: [13]). This protein family consists of pro-survival proteins like BCL-2, MCL1 and BCL-xL, initiator BCL2 homology- (BH3)-only proteins like PUMA, BIM and NOXA, and the cell death mediator proteins like BAX and BAK. The proteins of the BCL-2 family interact with each other by dimerization and apoptosis is initiated by BAX and BAK homo- or heterodimers. To prevent apoptosis, the pro-survival members like BCL-2 form heterodimers with BAX and BAK [19]. Stress signals like DNA damage lead to the activation of BH3-only proteins which bind BCL-2 to liberate BAX and BAK and thus induce apoptosis [20].

BH3 mimetics are a new class of cancer therapeutics that mimic the binding of BH3-only proteins to the hydrophobic groove of anti-apoptotic proteins [21]. In turn, heterodimerization of anti-apoptotic proteins with pro-apoptotic proteins is prevented and pro-apoptotic proteins are able to dimerize and trigger cell death [22]. ABT-737 is a BH3 mimetic that binds to and inhibits BCL-2, BCL-xL and BCL-w with high affinity but has a lower binding to MCL1 [23]. Consistent with its mechanism of action, the ability of ABT-737 to kill cells is dependent on the presence of BAK or BAX in the cell [24]. ABT-737 and its orally available analogue ABT-236 [25] already showed preclinical efficacy in some tumor cell models and are currently tested in clinical studies [26–30].

Based on the importance of BCL-2 proteins for apoptosis and for the therapy resistance of cancer cells, we studied the effect of the BH3 mimetic ABT-737 on proliferation and cell death induction in different thyroid carcinoma cell lines alone and in combination with chemotherapeutic drugs. With these experiments we verified the suitability of ABT-737 as a potential new therapeutic option for dedifferentiated thyroid carcinomas.

## Results

### ABT-737 decreased viability of thyroid carcinoma cells

**Fig. 1:**
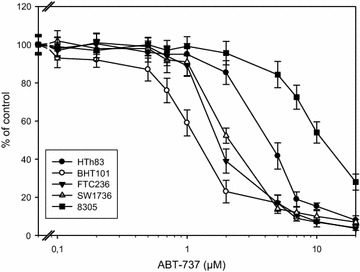
Decreased viability of thyroid carcinoma cells after ABT-737 incubation. Cells were cultured in the presence of increasing concentrations of ABT-737 or vehicle control for 48 h and viability was assessed by MTT assay. Values represent percent of vehicle control, mean ± standard deviation from eightfold determinations. IC50 values are shown in Table 1

Sixteen cell lines from FTC, PTC and ATC were treated with increasing concentrations of ABT-737 or vehicle for 48 h and the percentage of viable cells compared to controls was assessed. Treatment with ABT-737 decreased the number of cells in all 16 thyroid carcinoma cell lines analyzed, although to a variable degree (Table 1). IC50 values were in a relatively wide range of concentration (0.73–15.6 μM) with most values (12 of 16) between 1.0 and 5.0 µM. The lowest IC50 value of 0.73 μM was found in the B-CPAP PTC cell line, while three cell lines had IC values >10 μM (RO82W FTC cell line: 15.6 μM, 8305 and 8505 ATC cell lines: 10.9 and 10.1 μM; Table 1). IC50 values <2.0 μM were found in FTC and PTC cell lines. However, two cell lines derived from FTC (FTC238 and RO82W) depicted higher IC50 values (3.32 and 15.6 µM). In contrast, all IC50 values in ATC cell lines were >2.0 μM with SW1736, HTh7 and HTh74 cells being the most sensitive ATC cell lines (Table 1). As examples, results for FTC236, BHT101, SW1736, HTh83 and 8305 cells are shown in Fig. 1. Overall, the ABT-737 treatment decreased the quantity of viable cells in all 16 thyroid carcinoma cell lines examined. The IC50 values for ABT-737 treatment ranged between 0.73 and >10 μM with a slightly better effect in most of the more differentiated FTC and PTC cell lines.

**Table 1:** Origin and IC50 values of all thyroid carcinoma cell lines after 48 h of treatment with increasing concentrations of ABT-737 (MTT assay)

Cell line	Origin	IC50 ABT-737 (μM)
FTC133	FTC	1.31 ± 0.09
FTC236	FTC	1.71 ± 0.13
FTC238	FTC	3.32 ± 0.25
ML1	FTC	1.24 ± 0.12
TT2609	FTC	1.30 ± 0.10
RO82W	FTC	15.6 ± 1.12
BHT101	PTC	1.20 ± 0.09
B-CPAP	PTC	0.73 ± 0.08
TPC-1	PTC	1.79 ± 0.13
SW1736	ATC	2.05 ± 0.18
C643	ATC	4.89 ± 0.34
HTh7	ATC	2.39 ± 0.21
HTh74	ATC	2.27 ± 0.19
HTh83	ATC	4.10 ± 0.37
8305	ATC	10.9 ± 0.93
8505	ATC	10.1 ± 1.03

### Cell cycle analyses after ABT-737 treatment

**Fig. 2:**
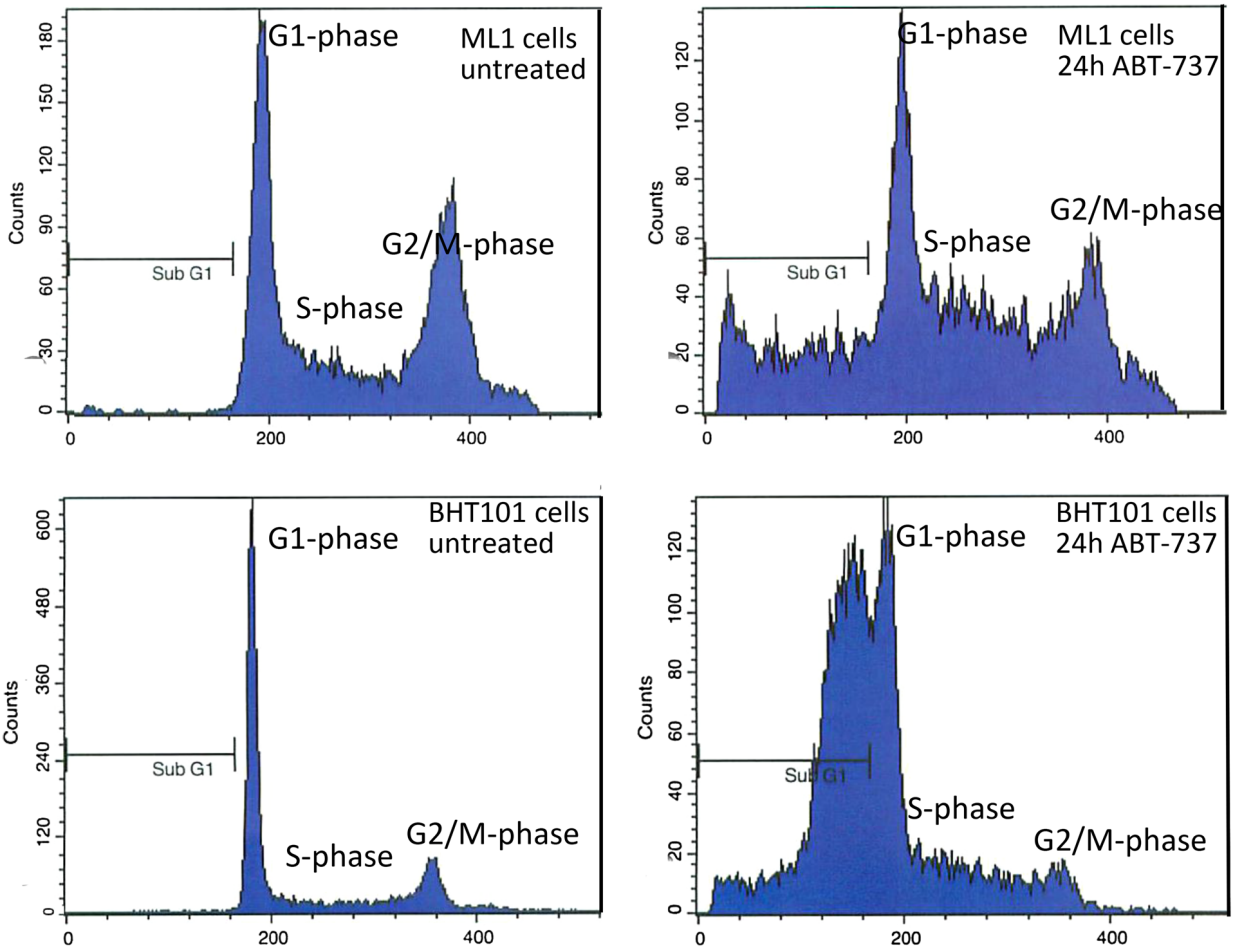
Cell cycle changes in ML1 and BHT101 cells after incubation with 1 µM ABT-737 for 24 h. Cell cycle analysis was conducted using FACS, results for ML1 and BHT101 cells are shown as examples. Besides the increase in SubG1 peak, in the remaining living cells an increase in S phase and a decrease in G1 and G2/M phase were observed. Values for all cell lines examined are shown in Table 2

Cell cycle analyses and the other experiments to determine the kind of cell death caused by ABT-737 were performed in five cell lines of different histological origin with similar IC50 values: ML1 and FTC236 cells were derived from FTC, BHT101 cells were derived from PTC and SW1736 and HTh7 from ATC. Cell cycle analyses of the propidium iodine stained DNA depicted a significant increase of cells in subG1 fraction in all five cell lines analyzed, pointing to ABT-737-induced cell death and DNA fragmentation (Table 2; Fig. 2). Interestingly, the percentage of cells in subG1 peak was the highest in ABT-737-treated papillary BHT101 and anaplastic SW1736 cells (54.8 and 39.9 %; Table 2). Both cell lines were shown to harbor a *BRAF*^*V600E*^ mutation that in thyroid tumors is found exclusively in carcinomas derived from PTC and which indicates that the ATC from which the SW1736 cells are derived originated as a PTC [31, 32]. Follicular ML1 and FTC236 cells and the anaplastic HTh7 cell line showed significantly increased values for the percentage of cells in subG1 peak of around 20 % after ABT-737 treatment (21.2; 18.8 and 20.1 %; Table 2). The remaining living cells from all five cell lines depicted a significant increase in the percentage of cells in the S phase of the cell cycle with 37.1–44.5 % of all living cells resting in S phase, while the percentage of cells in the G1 and G2/S-phase was diminished (Table 2).

**Table 2:** Distribution of cell cycle phases in vehicle-treated and ABT-737-treated thyroid carcinoma cells (24 h, 1 µM)

Cell line	Treatment	% subG1	% G1	% G2/M	% S
ML1 (FTC)	Untreated	0.39 ± 0.1	37.5 ± 2.1	30.5 ± 2.4	32.0 ± 2.2
	24 h ABT-737	21.2 ± 2.9*	34.0 ± 3.1	21.5 ± 1.9*	44.5 ± 3.6*
FTC236 (FTC)	Untreated	0.21 ± 0.1	48.5 ± 4.4	24.1 ± 1.6	27.4 ± 2.0
	24 h ABT-737	18.8 ± 2.0*	41.6 ± 3.9	20.3 ± 2.6	38.1 ± 2.9*
BHT101 (PTC)	Untreated	3.16 ± 2.8	65.1 ± 5.3	16.0 ± 1.7	18.9 ± 2.3
	24 h ABT-737	54.8 ± 4.6*	42.6 ± 5.3*	16.1 ± 2.7	41.3 ± 5.0*
SW1736 (ATC)	Untreated	1.52 ± 0.4	54.9 ± 5.4	15.2 ± 2.2	29.9 ± 2.6
	24 h ABT-737	39.9 ± 5.9*	42.8 ± 4.5*	13.2 ± 1.2	44.0 ± 3.6*
HTh7 (ATC)	Untreated	3.10 ± 0.2	60.1 ± 4.9	10.6 ± 0.8	29.3 ± 2.6
	24 h ABT-737	11.1 ± 1.4*	59.6 ± 4.7	3.3 ± 0.4*	37.1 ± 2.6*

Values for G1-, G2/M- and S-phase are determined for the living cells that were not included in the sub-G1-peak

* Indicates significant change (p < 0.05, Student’s t test) compared to controls treated with vehicle

### Cell death after ABT-737 treatment

**Fig. 3:**
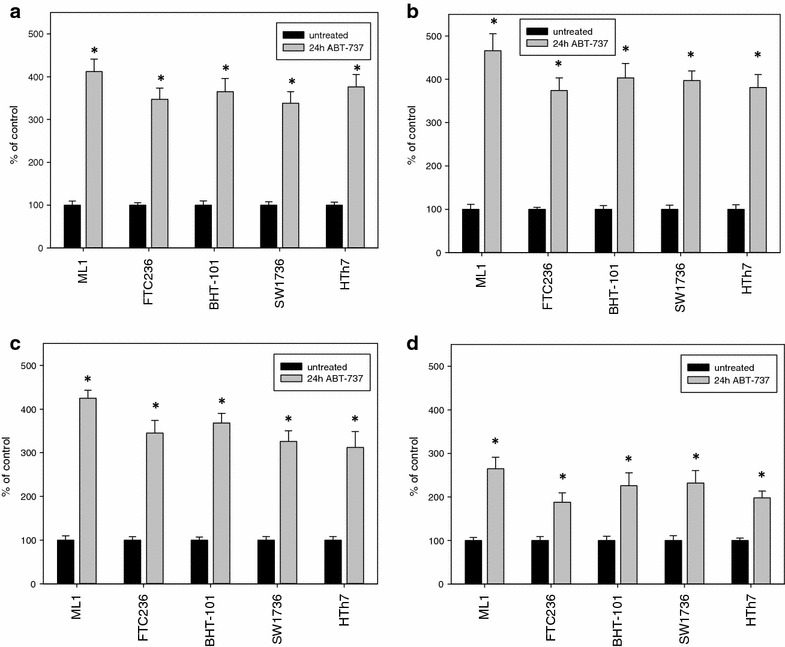
Increases in apoptosis markers and LDH release after treatment with 1 µM ABT-737 for 24 h. Caspase 3 and 7 activities (**a**) were determined by the ApoOne assay, cleaved caspase 3 (**b**) and cleaved PARP (**c**) concentrations were determined by specific ELISAs, and values for LDH-release into the medium (**d**) were determined by CytoTox-assay. All values are depicted as percent of vehicle-treated control, mean ± standard deviation from eightfold determinations; *indicates significant increase (p < 0.05, Student’s t test)

The kind of cell death induced by ABT-737 was analyzed biochemically in the five cell lines. To prove apoptotic cell death mechanisms after ABT-737 treatment, caspase 3 and 7 activity measurements and the increases in cleaved caspase 3 and cleaved PARP as products of activated caspases were analyzed. Caspase activities were significantly elevated after 24 h of ABT-737 treatment in all five thyroid carcinoma cell lines examined (Fig. 3a). ML1 cells exhibited the highest increase (412 % of control after 24 h), while in FTC236, BHT101, SW1736 and HTh7 cells, the increase in caspase 3/7 activities were between 338 % (SW1736) and 376 % (HTh7) of vehicle-treated control. Significant increases in cleaved caspase 3 (Fig. 3b) and cleaved PARP (Fig. 3c) as results of activated caspases were verified by specific ELISA analyses in all ABT-737-treated cells. Both increases were of the same magnitude in all five cell lines (374–466 % of control for cleaved caspase 3, Fig. 3b and 312–425 % of control for cleaved PARP, Fig. 3c) with ML1 cells being the most sensitive cell line. Moreover, LDH activity in supernatants of ABT-737-treated cells was significantly elevated in all five cell lines (188–265 % of control, Fig. 3d) indicating cell death by a disruption of cell membranes by necrosis or secondarily to apoptosis or other kinds of cell death. Taken together, our results of the DNA fragmentation depicted as SubG1 peak in cell cycle analyses, together with the increase in caspase activation after ABT-737 treatment pointed to an activation of the apoptosis machinery in treated cells.

**Fig. 4:**
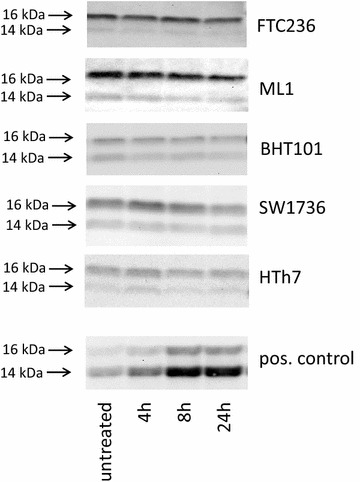
Absence of conversion of LC3B-I after treatment with 1 µM ABT-737 indicating no involvement of autophagic processes in ABT-737-mediated cell death. Western blot analyses of vehicle-treated and ABT-737-treated thyroid carcinoma cell lines using LC3B antibody are shown. HepG2 cells treated with 0.2 µM obatoclax were included as a positive control for autophagy induction. Equal protein loading was ensured by in-gel protein staining (see “Methods” section)

Furthermore, LC3B conversion as a marker of autophagic cell death was examined by western blot analyses after ABT-737 treatment to exclude the involvement of autophagic processes. As expected, all five thyroid carcinoma cells showed no LC3B cleavage while HepG2 cells treated with obatoclax which were used as positive control [33] depicted a clear conversion of LC3B isoforms (Fig. 4).

### Synergistic action of ABT-737 with chemotherapeutic agents

The effects of a combined treatment of thyroid carcinoma cells with ABT-737 and different chemotherapeutic agents (doxorubicin, gemcitabine, and cisplatin) on the survival rates of five thyroid carcinoma cell lines were investigated to study the possibility of a synergistic effect. The results are shown in Table 3. Calculation of survival rates according to the method of Drewinko et al. [34] yielded synergistic effects (Cl values >1.05) in three of five cell lines (FTC236, ML1, SW1736) for the combination of ABT-737 with doxorubicin, while BHT101 and HTh7 cells showed additive effects of both substances (Table 3a). For the combination of ABT-737 with gemcitabine, in four out of five cell lines synergism was observed, while papillary BHT101 only showed an additive effect (Table 3b). In combination with cisplatin, ABT-737 acted additively in all five cell lines (Table 3c). No antagonistic effect was observed (Table 3).

**Table 3:** Interaction of ABT-737 (0.5 µM) with doxorubicin (1.0 µM a), gemcitbine (0.5 µM b) or cisplatin (5.0 µM c) in five thyroid carcinoma cell lines

(a) Cell line	ABT-737	Doxorubicin	ABT-737 + doxorubicin	CI
FTC236 (FTC))	96.2 ± 6.5	92.4 ± 4.2	68.4 ± 5.9	1.30 syn.
ML1 (FTC)	86.5 ± 7.3	75.9 ± 5.4	37.6 ± 1.5	1.75 syn.
BHT101 (PTC)	84.4 ± 5.5	90.1 ± 6.1	78.6 ± 6.7	0.97 add.
SW1736 (ATC)	95.6 ± 7.0	74.3 ± 4.8	34.7 ± 3.0	2.05 syn.
HTh7 (ATC)	98.6 ± 4.6	65.0 ± 5.5	62.2 ± 3.1	1.03 add.

(b) Cell line	ABT-737	Gemcitabine	ABT-737 + gemcitabine	CI
FTC236 (FTC)	99.1 ± 4.8	79.4 ± 4.8	61.3 ± 5.0	1.28 syn.
ML1 (FTC)	86.0 ± 5.3	83.2 ± 5.4	54.8 ± 3.8	1.31 syn.
BHT101 (PTC)	84.9 ± 6.9	87.6 ± 6.7	76.2 ± 5.2	0.98 add.
SW1736 (ATC)	96.8 ± 6.1	78.5 ± 3.8	56.1 ± 2.5	1.35 syn.
HTh7 (ATC)	97.3 ± 7.5	71.8 ± 4.2	42.9 ± 3.9	1.63 syn.

(c) Cell line	ABT-737	Cisplatin	ABT-737 + cisplatin	CI
FTC236 (FTC)	95.9 ± 7.9	102.6 ± 9.3	99.7 ± 7.4	0.99 add.
ML1 (FTC)	84.8 ± 5.8	97.3 ± 7.4	83.5 ± 6.9	0.99 add.
BHT101 (PTC)	83.4 ± 6.3	98.1 ± 8.7	81.7 ± 5.7	1.00 add.
SW1736 (ATC)	95.0 ± 5.6	103.8 ± 7.2	101.9 ± 6.9	0.97 add.
HTh7 (ATC)	99.3 ± 7.8	98.5 ± 5.9	100.2 ± 7.0	0.98 add.

MTT assays were performed to determine the viability of cells after incubation with one compound alone or in combination. Cl values were calculated according to the method of Drewinko et al. [34; see “Methods” section], where Cl > 1.05 indicates synergism (syn.), 0.95 ≤ Cl ≤ 1.05 indicates additivity (add.) and Cl < 0.95 indicates antagonism

## Discussion

With our experiments we showed the efficacy of the BH3 mimetic drug ABT-737 in reducing the number of viable cells and in inducing apoptotic cell death in thyroid carcinoma cells of various histological origins.

Patients with dedifferentiated and anaplastic thyroid carcinomas are difficult to treat since during dedifferentiation thyroid carcinoma cells lose their ability to take up radioiodine. Furthermore, thyroid carcinoma cells are resistant to external radiation and chemotherapeutic treatment [4]. The inability of these cells to undergo apoptotic cell death is one reason for this resistance [12, 13] and may be influenced by a new class of pharmacological compounds termed BH3 mimetics [23, 35]. BH3 mimetic drugs, such as ABT-737, are due to counter apoptotic blocks by binding to anti-apoptotic proteins like BCL-2, BCL-xL and BCL-w [23, 24]. In preclinical studies, ABT-737 showed single agent efficacy against some tumor types like small cell lung cancer, follicular lymphoma and chronic lymphocytic leukemia [36–38]. Some recently completed phase 1 trials with ABT-263, the orally available analogue drug of ABT-737, also showed clinical responses in 30–50 % of patients with chronic lymphocytic leukemia (CLL) [26, 27] but not in small cell lung cancer (SCLC) patients [28].

In this study we showed that ABT-737 is also active against dedifferentiated and anaplastic thyroid carcinoma cells by inducing apoptotic cell death. ABT-737 reduced the number of viable cells in all 16 thyroid carcinoma cell lines investigated. All IC50 concentrations measured, except that of B-CPAP cells (0.73 μM) were above 1 μM but in a relatively narrow range (up to 15.6 μM). IC50 values found in this study were thus in the range already reported for lymphoma [36], leukaemia [39] and glioblastoma cells [40]. However, IC50 values were higher than those of extremely sensitive primary CLL cells with IC50 values in the low nanomolar range [41] and in a subset of SCLC cell lines which exhibited IC50 values <0.1 μM [23, 37, 38].

Our biochemical data in ABT-737-treated thyroid carcinoma cells argue for an apoptotic cell death with activation of caspases, increase in caspase cleavage products and DNA fragmentation. An increased LDH release after ABT-737 treatment probably due to cell death by secondary necrosis was seen in all five cell lines examined. These data fit well with the proposed mechanism of action of ABT-737. It was shown that ABT-737 mimics the action of the BH3-only protein BAD by binding the anti-apoptotic proteins BCL-2, BCL-xL and BCL-w and inhibits its function so that apoptosis can be induced [23]. Induction of autophagy by ABT-737 by disrupting the interaction of BECLIN1 with BCL-2 and BCL-xL was also indicated in single reports [42] but was not seen in our thyroid carcinoma cells as no LC3B cleavage following ABT-737 incubation was depicted.

A better sensitivity towards ABT-737 was, with the exception of FTC cell lines FTC238 and RO82W, found in FTC and PTC cell lines compared to more dedifferentiated ATC cell lines reflecting the aggressiveness and treatment resistance of ATC cells. Regarding the sensitivity towards ABT-737, it was reported that ABT-737 is effective in cells with overexpressed BCL-2, BCL-xL or BCL-w but does not bind to the anti-apoptotic proteins MCL1 or A1 [23]. Accordingly, it has been shown that an elevated expression of MCL1 or BFL-1/A1 may mediate the resistance of tumor cells to ABT-737-mediated apoptosis [36, 43]. Furthermore, the effect of ABT-737 is dependent on the expression of the pro-apoptotic proteins BAK and/or BAX which execute apoptosis when liberated from BCL-2 after ABT-737 treatment [39, 44]. In thyroid tissues, an elevated expression of BAX was demonstrated in thyroid carcinoma compared to adenoma [45]. Furthermore it was shown in different studies that BCL-2 expression is strong in most differentiated thyroid carcinoma but decreases in less differentiated subtypes [46–49]. BCL-xL expression on the other hand was shown to be stronger in high-risk subtypes of thyroid carcinoma [50], while little is known about MCL1 expression in thyroid carcinoma tissues. Expression profiles of BCL-2 family proteins in our thyroid carcinoma cells was already analyzed in a recent study by our group [51]: While expression of BAK was relatively constant as a prerequisite for ABT-737 action, BAX was not expressed in FTC238 and C643 and showed a weak expression in sensitive BHT101 and insensitive 8305 and 8505 cells [51]. Expression of BCL-2, BCL-xL and MCL-1 was variable [51] but not related to ABT-737 sensitivity (Table 1): The most sensitive cell line B-CPAP (IC50: 0.73 μM) showed a weak expression of both BCL-2 and BCL-xL, while MCL-1 was moderately expressed [51]. The four most sensitive cell lines besides B-CPAP (BHT101, ML1, FTC133 and TT2609) on the other hand showed a strong expression of either BCL-2 or BCL-xL and moderate expression of MCL-1 [51]. The most insensitive cell line RO82W expressed BCL-xL strongly and BCL-2 moderately [51]. In sensitive BHT101 (IC50: 1.20 μM) as well as in insensitive 8305 cells (IC50: 10.9 μM), a very low expression of both BCL-2 and BCL-xL proteins was seen [51]. Furthermore, in contrast to recent data of Iacovelli et al. [52] who showed that in five acute lymphocytic leukemia (ALL) cell lines the ratio of MCL-1/BCL-2 plus BCL-xL protein ratio was correlated with sensitivity to ABT-737, in the 16 thyroid carcinoma cell lines analyzed in this study, no such correlation was found [51]. In mouse cell lines derived from thyroids of mouse strains with deleted *p53* and activated *Kras* that develop PTC and PDTC, high expression of *Bcl*-*2* and *Mcl1* was reported that mediate resistance to apoptosis [53]. These cell lines can be targeted by GX15-070 (obatoclax), a pan-inhibitor of the BCL-2 family, while ABT-263 was modestly effective [53] which generally showed the suitability of BH3 mimetics for treatment of thyroid carcinoma cells. In one early study, Mitsiades and coworkers [54] also showed the efficacy of the BH3 inhibitors BH3I-1 and BH3I-2 in some thyroid carcinoma cell lines as well as sensitization to other anti-tumor substances [54]. In own experiments, we have recently shown the potency of GX15-070 against dedifferentiated thyroid carcinoma cells of various histological origins [51]. Treatment with GX15-070 resulted in a non-classical cell death with signs of apoptosis, autophagy and necrosis in parallel [51] that was also seen in other cell systems [55–57]. While GX15-070 and its cellular targets besides the proteins of the BCL-2 family are not known yet, ABT-737 was shown to act as a real BH3 mimetic [23]. However, our data indicate that the expression of pro- and anti-apoptotic proteins alone does not predict sensitivity to ABT-737. These results are underlined by several other recent papers: In ovarian carcinomas, it was shown that phospho-ERK1/2 as well as a low expression of BIM are biomarkers for absence of response to ABT-737 [58]. Phosphorylation of MCL-1 and BCL-2 are found to be further determinants of sensitivity to ABT-737 [59, 60]. Phosphorylation of MCL-1 at various threonine and serine residues by Cyclin E/Cdk2 kinase, ERK (extracellular-signal regulated kinase), JNK (c-jun N-terminal kinase), p38 MAPK (mitogen-activated kinase) and GSK-3β (glycogen synthase kinase-3β) can lead to stabilization as well as destabilization of MCL-1 [59, 61–64], while phosphorylation of BCL-2 leads to a structural alteration in the BH3-binding groove and resistance to ABT-737 [60]. Furthermore, treatment of cells with ABT-737 can lead to altered expression of proteins of the BCL-2 family [65, 66]. Thus, prediction of sensitivity of a cell line to ABT-737 treatment is a topic under investigation in many cell systems and also needs further investigation in thyroid carcinoma cells. However, with the availability of ABT-737 and its orally active derivative ABT-263, our data on the potency of BH3 mimetics become a current topic.

Furthermore, facilitating cell death of cancer cells by simultaneous treatment with ABT-737 and chemotherapeutic drugs is a logical consequence of the mechanism of actions of both kinds of drugs. Since chemotherapeutic agents kill cells mainly via the mitochondrial apoptosis pathway [67] antagonists of BCL-2 proteins may influence and facilitate cell death induction by these agents. The five thyroid carcinoma cell lines examined for synergistic action of ABT-737 with chemotherapeutic agents exhibited different reaction patterns for these drug combinations: doxorubicine and gemcitabine were the most effective combinations with ABT-737 and induced synergistic effects in three or four cell lines. Papillary BHT101 cells in all combinations showed only additive effects, while cisplatin in combination with ABT-737 only had additive effects in all five cell lines (Table 3).

In other cell systems, ABT-737 was also shown to have synergistic action with other agents like cytotoxic drugs in inducing cell death like carboplatin against ovarian cancer and SCLC [37, 68], etoposide against SCLC [38], docetaxel against breast cancer [69] as well as kinase inhibitors like PI3 kinase and mTOR inhibitors in SCLC and colorectal cancer cells [70, 71]. The sensitizing and apoptosis-facilitating effects for chemotherapeutic drugs by ABT-737 demonstrated here may, beyond the use of ABT-737 as single drug, be of interest as a further therapeutic option in dedifferentiated thyroid carcinomas.

## Conclusion

In summary, we found that ABT-737 was effective in dedifferentiated and anaplastic thyroid carcinoma cells by inducing apoptotic cell death and furthermore showed synergistic effects with doxorubicin and gemcitabine. Although not all of the 16 cell lines examined were sensitive towards ABT-737 to the same extend, ABT-737 and related BH3 mimetics may have value as new therapeutic options for dedifferentiated thyroid cancer as monotherapy or in combination with other anticancer agents.

## Methods

### Compounds and antibodies

ABT-737 and obatoclax were from Selleck Chemicals (Houston, TX, USA). They were stored in 10 mM aliquots in DMSO at −20 °C and further diluted in the appropriate medium. Chemotherapeutic agents were from Merck-Millipore (Darmstadt, Germany).Antibodies were from Cell Signaling Technology (Danvers, MA, USA).

### Cell lines and cell culture

Established thyroid cell lines from ATC, PTC and FTC were used in this study. The SW1736, HTh7, HTh74, HTh83, C643, 8305 and 8505C cell lines were derived from ATC. BHT101, B-CPAP and TPC1 were derived from PTC. ML1, RO82W, and TT2609 are FTC cell lines. The FTC133, FTC236 and FTC238 cell lines [72] were derived from a single primary FTC, a lymph node metastasis and a lung metastasis from the same patient. HepG2 hepatocellular carcinoma cells were used as control for induction of autophagy. The HTh7 [73], HTh74 [74], HTh83 [75], C643 [76] and SW1736 [77] cell lines were a gift from Prof. Heldin (Uppsala, Sweden), and all other cell lines were purchased from ATCC (Manassas, VA, USA), ECACC (Salisbury, UK) and DSMZ (Braunschweig, Germany). Cell lines were maintained in their appropriate media supplemented with 10 % fetal bovine serum (FBS, Life Technologies, Paisley, PA, USA) at 37 °C at 5 % CO_2_.

### Cell survival studies

Depending on the cell line, 1 × 10^4^–5 × 10^4^ cells were seeded into 96-well plates. Medium was removed after 24 h and replaced with culture medium without FBS but containing 0.1 % bovine serum albumin (BSA) and the indicated concentrations of ABT-737, chemotherapeutic agents or a combination of both as indicated. After 48 h, viable cells were stained with the Cell Titer Aqueous One Solution assay (Promega, Madison, WI, USA). Optical density at 490 nm was measured with an Emax microplate photometer (Molecular Devices, Sunnyvale, CA, USA). Control values without treatment were performed as 22-fold determinations, while all concentrations of ABT-737, chemotherapeutic agents and combinations were tested in eightfold. Calculation of results and Student’s t test were performed using SoftMax pro software (Molecular Devices), and IC50 values were calculated using Sigma Plot software (Systat, San Jose, CA, USA). Interaction of ABT-737 with chemotherapeutic agents was calculated according to the method of Drewinko et al. [34] using the following formula: Cl = (survival a × survival b)/(survival (a + b) × 100), where a and b indicate the two drugs used and a + b indicates the combination of a and b. The interaction was interpreted on the basis of Cl values where Cl > 1.05 indicates synergism, 0.95 ≤ Cl ≤ 1.05 indicates additivity and Cl < 0.95 indicates antagonism.

### Measurement of lactate dehydrogenase (LDH) release and caspase 3/7 activity

Measurement of lactate dehydrogenase (LDH) released from cells with damaged membranes was performed by the CytoTox-ONE homogeneous membrane integrity assay (Promega). Determination of caspases 3 and 7 activity was done by the ApoONE homogeneous Caspase 3/7 assay (Promega). 1 × 10^4^–5 × 10^4^ cells (cell line dependent) were seeded into black, transparent-bottomed 96-well plates in their appropriate growth medium. Medium was removed after 24 h and 100 μl culture medium with 0.1 % BSA but without FBS that contained the denoted ABT-737 concentration, was added to each well. After 24 h, 50 μl of medium from each well was transferred to a fresh black 96-well plate and equilibrated to 20 °C. Fifty microliter of CytoTox reagent was added, and reactions were incubated for 10 min in the dark at room temperature. Twenty five microliter of stop solution was added and fluorescence was determined with an excitation wavelength of 560 nm and an emission wavelength of 590 nm, respectively. In each experiment, wells containing no cells as the zero setting, and fully lysed cells as the maximum LDH release control, were included. Activity of caspase 3 and 7 was determined in the original stimulation plate by adding 50 μl of ApoONE reagent that contained a fluorometric caspase substrate in cell lysis and reagent buffer. After 60 min, fluorescence was measured at 521 nm after excitation with 499 nm. All values were performed as eightfold determinations. Calculation of results and Student’s t tests were performed using SoftMax pro software (Molecular Devices).

### Cell cycle analysis

1 × 10^5^–5 × 10^5^ cells per well were plated in six-well plates in their appropriate growth medium. Medium was replaced with medium without FBS but containing 0.1 % BSA and 1 μM ABT-737 or vehicle after 24 h and cells were incubated for 24 h. Treated cells were harvested and fixed in cold 70 % ethanol. RNase A (60 μg/ml) and propidium iodide (25 μg/ml) in PBS were added, and samples were incubated 20 min in the dark at RT. Fluorescence was measured on a FACS Calibur flow cytometer (Becton–Dickinson, San Jose, CA), and cell cycle stages were analyzed using the ModFit Software (Verity Software House, Topsham, ME, USA).

### Cell stimulation and protein extraction

For ELISA and western blot analyses, cells were seeded on cell culture dishes and grown for 1–2 days until they reached 80–85 % confluence. Medium was replaced with medium containing 0.1 % BSA and cells were maintained in this medium for 1 h before 1 μM ABT-737 or vehicle was added. For positive control of autophagy, HepG1 cells were incubated with 0.2 μM obatoclax (GX15-070). After the indicated times, the medium was removed and collected and the cells were scraped in PBS. For cell lysis, a lysis buffer containing protease inhibitors (Complete protease inhibitor, Roche Applied Science, Mannheim, Germany) was used. The lysates were centrifugated at 10,000*g* for 10 min at 4 °C. The protein concentration was determined with a modified Bradford assay (Bio-Rad Laboratories, Hercules, CA, USA).

### Cleaved caspase and cleaved PARP ELISA

Specific sandwich ELISAs were used to determine cleaved caspase 3 (Asp175) and cleaved poly (ADP ribose) polymerase (PARP) as a marker of apoptosis induction and protease activation (Cell Signaling Technologies) [78, 79]. In brief, cells were plated, stimulated, and lysed as described above. Hundred microliter of diluted cell lysate containing 100 μg of total protein was incubated in each of the antibody-coated well of the plate overnight at 4 °C. After washing, an antibody specific for the cleaved protein and a HRP-labelled secondary antibody for detection were used. Substrate reaction was started by addition of TMB and was stopped after 30 min room temperature. Absorbance was determined at 450 nm (EMax microplate reader). The results were calculated as percent of unstimulated controls using SoftMax pro software (Molecular Devices).

### Western blot analyses

The effects of incubation with ABT-737 or with obatoclax as the positive control on microtubule-associated protein 1A/1B-light chain 3 (LC3B) cleavage as a marker of autophagic cell death was analysed by western blot. 30 μg of total protein from treated and vehicle-treated cells (see above) were denatured by boiling for 5 min in SDS sample buffer. Proteins were separated by SDS-PAGE on stain-free polyacryl amide gels (Bio-Rad Laboratories) to enable loading control [80, 81]. After electrophoresis, optical densities of stained total proteins in each lane were documented with a CCD camera system and verified using the Quantity One software (both Bio-Rad Laboratories). When the integrated optical densities of proteins in each lane did not differ more than 10 %, proteins were transferred to a nitrocellulose membrane (Bio-Rad Laboratories). The blots were blocked with BSA and incubated with the LC3B primary antibody (Cell Signaling Technologies) in TBS containing 0.1 % Triton X100 overnight at 4 °C. An appropriate secondary antibody coupled to horseradish peroxidase was added and detection of bound antigens was performed by an enhanced chemiluminescence detection kit (Amersham ECL Advance, GE Healthcare, Piscataway, NJ, USA). Signal intensity was evaluated with a CCD-camera (Bio-Rad Laboratories).

### Statistical analysis

Statistical analysis of treatment versus control groups was performed by means of the unpaired Student’s t test using SPSS (IBM Inc, Armonk, NY, USA) or the other software packages indicated above. P values <0.05 were considered statistically significant.

## Abbreviations

ALL: acute lymphocytic leukemia
ATC: anaplastic thyroid carcinoma
BCL-2: B-cell lymphoma
BH3: BCL-2 homology
CLL: chronic lymphocytic leukemia
ERK: extracellular-signal regulated kinase
FTC: follicular thyroid carcinoma
GSK-3β: glycogen synthase kinase-3β
JNK: c-jun N-terminal kinase
LC3: microtubule-associated protein 1A/1B-light chain 3
LDH: lactate dehydrogenase
MAPK: mitogen-activated kinase
PARP: poly-(ADP-ribose)-polymerase
PDTC: poorly diferentiated thyroid carcinoma
PTC: papillary thyroid carcinoma
SCLC: small cell lung cancer
